# A simple minimally invasive technique of removing lumbar disc debris following discectomy

**DOI:** 10.1308/003588412X13171221591259g

**Published:** 2012-05

**Authors:** NK Patel, RA Bajekal

**Affiliations:** Barnet and Chase Farm Hospitals NHS TrustUK

## BACKGROUND

Lumbar disc herniation is common and can be treated with a dis- cectomy. Open discectomy usually involves a posterior incision and laminotomy to access the vertebral canal. An obvious disc herniation can be removed piecemeal using pituitary forceps and an annulotomy allows access inside the disc. Removal requires work through a small incision at a depth that can be challenging and the anterior annulus must be left intact to avoid damaging vascular structures. Large fragments of disc may therefore often be left behind unintentionally, resulting in a recurrent disc herniation rate of 7–12%^1^,^2^ and up to 38% of patients having persistent sciatica.^3^

**Figure 1:**
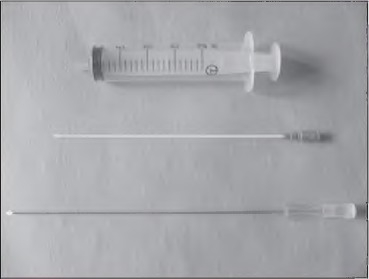
Abbocath®-T catheter (sheath and needle) with a 20ml syringe

## TECHNIQUE

Once much of the disc has been removed manually using pituitary forceps, a 16G X 140mm Abbocath®-T radiopaque fluorinated ethylene propylene intravenous catheter (Hospira Inc, Lake Forest, IL, US) is inserted into the disc via the annulotomy. The inner needle is removed and a 20ml syringe filled with normal saline solution is attached to the Abbocath®-T catheter. This is injected at pressure into the centre of the disc, following the path of least resistance. This helps break up parts of residual disc, causing extrusion of debris through the annulotomy, where it can be removed easily with the pituitary forceps. The procedure can be repeated until the effluent solution is clear and there is no resistance to flow.

## DISCUSSION

This is a simple and effective technique for removing debris following an open lumbar discectomy. Furthermore, it is safe and may lower the risk of recurrent disc herniation although further studies are indicated.
